# Immunologic Dose-Response to Adenovirus-Vectored Vaccines in Animals and Humans: A Systematic Review of Dose-Response Studies of Replication Incompetent Adenoviral Vaccine Vectors when Given via an Intramuscular or Subcutaneous Route

**DOI:** 10.3390/vaccines8010131

**Published:** 2020-03-17

**Authors:** Sara Afrough, Sophie Rhodes, Thomas Evans, Richard White, John Benest

**Affiliations:** 1 Vaccitech Ltd., The Schrodinger Building, Heatley Road, The Oxford Science Park, Oxford OX4 4GE, UK; tom.evans@vaccitech.co.uk; 2 Department of Infectious Disease Epidemiology, London School of Hygiene and Tropical Medicine, Keppel Street, London WC1E 7HT, UK; Sophie.Rhodes@lshtm.ac.uk (S.R.); Richard.White@lshtm.ac.uk (R.W.); John.Benest@lshtm.ac.uk (J.B.)

**Keywords:** dosing, dose-response, adenovirus-vectored vaccines, immunogenicity, clinical, pre-clinical

## Abstract

Optimal vaccine dosing is important to ensure the greatest protection and safety. Analysis of dose-response data, from previous studies, may inform future studies to determine the optimal dose. Implementing more quantitative modelling approaches in vaccine dose finding have been recently suggested to accelerate vaccine development. Adenoviral vectored vaccines are in advanced stage of development for a variety of prophylactic and therapeutic indications, however dose-response has not yet been systematically determined. To further inform adenoviral vectored vaccines dose identification, historical dose-response data should be systematically reviewed. A systematic literature review was conducted to collate and describe the available dose-response studies for adenovirus vectored vaccines. Of 2787 papers identified by Medline search strategy, 35 were found to conform to pre-defined criteria. The majority of studies were in mice or humans and studied adenovirus serotype 5. Dose-response data were available for 12 different immunological responses. The majority of papers evaluated three dose levels, only two evaluated more than five dose levels. The most common dosing range was 10^7^–10^10^ viral particles in mouse studies and 10^8^–10^11^ viral particles in human studies. Data were available on adenovirus vaccine dose-response, primarily on adenovirus serotype 5 backbones and in mice and humans. These data could be used for quantitative adenoviral vectored vaccine dose optimisation analysis.

## 1. Introduction

The methods of finding doses for optimal vaccine delivery in humans is an empirical science. Frequently, vaccine developers have relied on historic information to conduct small dose-ranging studies in animal models, and then used these data to design further studies in humans, despite the relationship between animal and human dose being unproven [1]. Unlike allometric analysis used in pharmacokinetic/pharmacodynamic assessments in small molecule drug development [2], there are no published or widely accepted allometric scaling factors to easily translate animal vaccine dose-responses to human vaccination. Thus, each vaccine development group collates the relevant literature, and their own data, to determine how to design initial animal or human dose-response studies. Unfortunately, recent evidence suggests that this empirical method of dose selection has, in part, led to suboptimal dose identification in humans for diseases, such as yellow fever, meningitis and malaria [3,4,5,6].

Recently developed mathematical modelling methods, referred to as immunostimulation/immunodynamic (IS/ID) modelling, attempts to address these issues [7,8,9,10]. IS/ID modelling was developed to address the lack of quantitative methods in vaccine development [11]. The aim of IS/ID is to translate pharmacokinetic/pharmacodynamic (PK/PD) methodology to vaccine development, and preliminary IS/ID modelling has shown promise in accelerating vaccine dosing decisions. Modelling of the dose-response curve and cross-species translation of tuberculosis vaccine dosing data have predicted a lower human dose than previously tested [7,9], and showed that antibody response against human parainfluenza virus may be maximised by an intermediate dose [12]. To inform future IS/ID modelling, dose-response data must be collated. However, these data can also provide valuable insight into study designs that are currently used to explore vaccine dose-response, as understanding the scope of previous dose-ranging trials may be of use in determining the cause of suboptimal dosing.

Adenovirus vectored vaccines have been widely investigated for their ability to induce antibody and T cell responses against infectious diseases and cancers [13]. However, the dose-response for adenoviral vectored vaccines has not yet been systematically investigated. In this systematic review, we aim to explore and collate available adenoviral dose-response data for the purpose of informing adenoviral dosing towards safer and more effective vaccination. Our objectives were to:

1. Assess the number of available papers, including adenoviral dose-response studies, and the distribution of host species and adenoviral serotypes within these papers.
2. Assess which immunological responses dose-response data were available.
3. Assess the dosing strategies used in adenoviral dose-ranging studies, including number and magnitude of dose levels.

This systematic review should help inform adenoviral vaccine developers in choosing dose amounts for first-in-human trials. The collated data on dose-response, and replicating incompetent adenovirus-based vaccines, will also be used to inform IS/ID modelling studies for vaccine dose optimisation.

## 2. Materials and Methods

The study protocol was registered in PROSPERO (CRD42017080183).

### 2.1. Study Types, Study Design, Population, Intervention and Outcome Measures

Papers on clinical trials and in-vivo pre-clinical studies, that presented data from adenovirus vector-induced immunogenicity, were included in the review. These could include data from humans and animals of any age, sex and genetic background who received adenoviral vectored vaccines administered intramuscularly or subcutaneously. We did not assess study design aspects, such as methods of randomisation or use of control groups. The primary outcome measures were humoral and cellular immunity.

### 2.2. Search Strategy

The MEDLINE (PubMed) database was searched from inception to 27 November 2018. The search was limited to papers published in English and included terms relating to the following concepts: Adenovirus-vectored vaccines, immunogenicity, and dose-response (Appendix A, Criteria A1).

### 2.3. Paper Selection (Inclusion/Exclusion Criteria)

**Figure A1:**
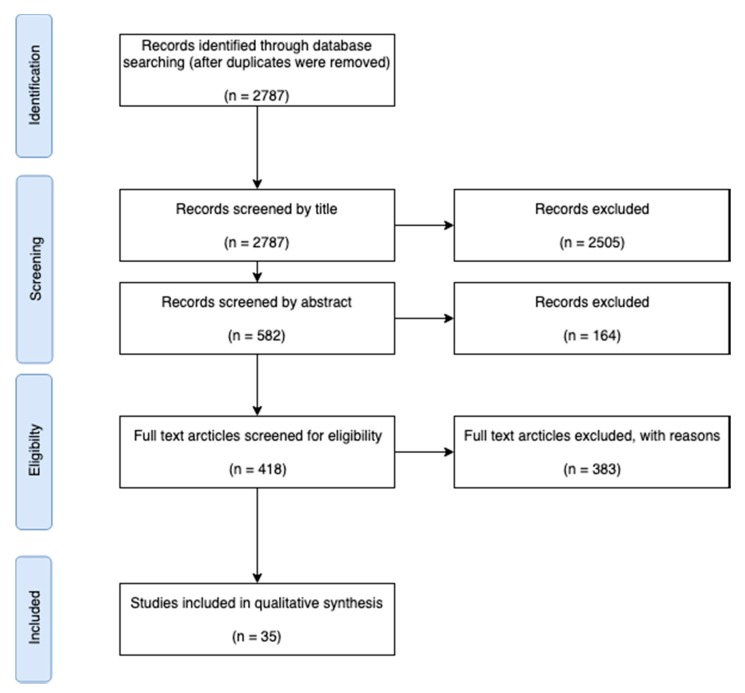
Flow chart diagram of the search and screening strategy.

A three-stage screening process was used to systematically screen retrieved references and assess whether they met the inclusion criteria. Papers were first screened by title then by abstract before a full-text screen was conducted (Appendix A, Figure A1).

**Figure A2:**
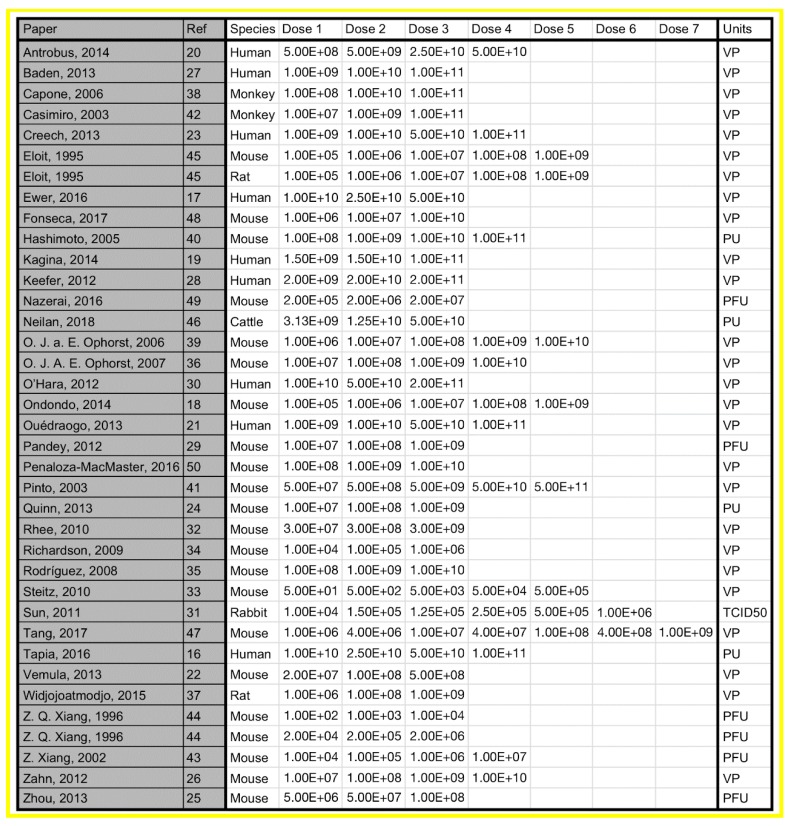
Dose magnitudes for the collected studies, including the name of the paper, the host species, and the unit used to quantify dose.

We included papers that presented data from studies with immunological response at three or more dose levels of an adenoviral vectored vaccine. We included papers that captured CD4+/CD8+ T-cell response, as measured by cytokine release using either ELISPOT or multiparameter flow cytometry and/or humoral responses, including binding and neutralising antibody titres against the vector and antigen. Exclusion criteria were chosen to minimize the probability of response being altered by a non-dosal effect, for example excluding cancer models and prime-boost paradigm vaccination (Appendix A, Figure A2).

### 2.4. Data Extraction

Using a pre-designed data extraction spreadsheet, information relating to study characteristics were extracted from studies that met the inclusion criteria. Numerical data from figures were extracted using GraphClick version 2.9.2 (Arizona Software, Los Angeles, CA, USA). Papers could contain data from multiple dose-response studies, and these studies may vary in adenoviral serotype, route of administration, host species, or disease.

### 2.5. Assessment of Methodological Quality

Bias was controlled for by having two individuals participate in the original search, and on abstract review. A review of 10 articles known to be relevant was conducted, to evaluate the degree of completeness. No statistical methods were performed to assess publication bias.

### 2.6. Comparing Doses

Three different units of measurement of dose were used in the extracted studies; viral particles (VPs), particle units (PUs), or plaque forming units (PFUs). Doses measured in VPs and PUs were considered equivalent as they both measure the number of physical viral particles [14]. PFUs were considered a separate outcome, as the ratio of VPs/PUs to PFUs were not constant in adenoviral vaccines studies [15].

## 3. Results

### 3.1. Objective 1: Assess the Number of Available Papers Including Adenoviral Dose-Response Studies, and the Distribution of Host Species and Adenoviral Serotypes within These Papers

Following removal of duplicate entries, 2787 references remained and were screened by title. 581 references were screened by abstract and 300 were screened by full text. After evaluation of the full text, 265 of the papers were excluded. Therefore, 35 papers were included in this review [16,17,18,19,20,21,22,23,24,25,26,27,28,29,30,31,32,33,34,35,36,37,38,39,40,41,42,43,44,45,46,47,48,49,50]. The majority of papers contained studies conducted in mice (60%), followed by humans (26%) (Table 1). Although, it is likely that many studies may have been carried out by industry using the same construct in mice and humans, the number of published studies using the same adenoviral strain, route and antigen insert across different species was limited.

Out of all the adenoviral serotypes, the most common was human adenovirus 5 (46%), followed by human adenovirus 35 (26%) (Table 2). The route of administration was more frequently intramuscular (84%) than subcutaneous (16%).

### 3.2. Objective 2: Assess for Which Immunological Responses Dose-Response Data Were Available

The immunogenicity data recorded also varied widely among the published studies, including antibody responses (both binding and neutralizing), T cell ELISpot data, and CD4+ and CD8+ T cell responses by intracellular cytokine staining. The majority of papers (51%) included studies of antibody dose-response. (Table 3).

### 3.3. Objective 3: Assess the Dosing Strategies Used in Adenoviral Dose-Ranging Studies, Including Number and Magnitude of Dose Levels

#### 3.3.1. Number of Dose Levels

The majority of papers (60%) included studies with three dose levels, which was the minimum number of dose levels for a study to be included; 23% included four dose levels, and 20% included five or more levels (Table 4).

#### 3.3.2. Magnitude of Dose Levels

**Figure 1:**
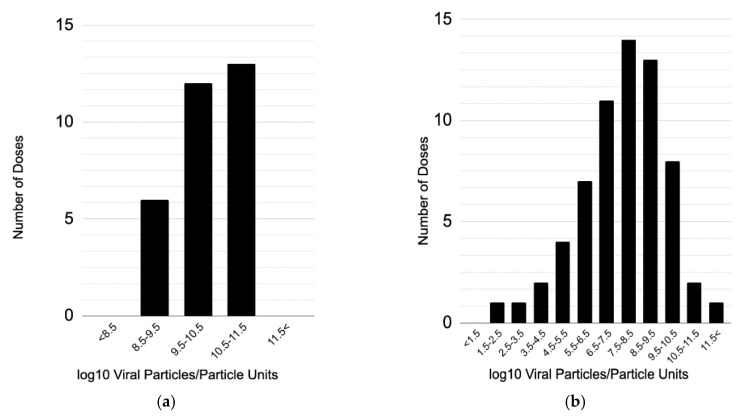
Frequency of dose magnitudes for all adenoviral vector vaccine dose-response studies with doses measured in VP/PFU in (**a**) humans and (**b**) mice.

For VP/PU, the geometric mean human dose was 1.6 × 10^10^ (range 5 × 10^8^–2 × 10^11^) (Figure 1a). No human dose-response studies were measured in PFU. In VP, the geometric mean mouse dose was 4.9 × 10^7^, (range 5 × 10^1^–5 × 10^11^) (Figure 1b). The mean human dose was therefore approximately 3.2 × 10^2^ times larger than the mean mouse dose. Four mouse dose-response studies measured dose in PFU, with doses ranging between 1 × 10^4^ and 1 × 10^9^ PFU. Details on the magnitude of dose levels are found in Appendix A, Figure A2.

## 4. Discussion

In this review we aimed to collate data on adenovirus-based vaccines in preparation for mathematical modelling to characterize the dose-response curve by host species, serogroup of the adenovirus or route of administration. After screening, 35 papers were extracted that provided dose-response immunogenicity data following intramuscularly or subcutaneously administered adenovirus vectors in animals and humans. Data were primarily from mouse and human studies, and included multiple different response types. From the adenoviral dose-response papers considered, studies typically used three dose levels, with the average human dose being two orders of magnitude larger than the average mouse dose. There were unfortunately very few comparator trials in which the same vaccine was used in human and animal models, and much of the pre-clinical data from larger industry companies are unlikely to have been published.

This review represents the first attempt to collate vaccine dose-response data, which has not yet been done for adenoviral vectored or non-adenovirus vectored vaccines. The review found that dose-response data existed for a wide range of immunological responses, both humoral and cellular. This suggests that published dose-response data may exist for many important correlates of protection. The broad spectrum of available data will be used to inform an IS/ID modelling study on adenoviral dose-response curve shape. However, the majority of studies used too few doses to allow for true dose response relationships to be clearly established, and thus, the majority of studies conducted are not sufficient to allow the authors to clearly justify their dose selection. To establish a true sigmoidal curve fit, at least five data points are needed to accurately model the response.

Whilst this review was able to identify 35 papers that may be useful in understanding adenoviral vectored vaccine dose-response behaviour, there are factors that may limit the utility of the collated data. Firstly, it is likely that there exist vaccine dose-response studies that have not been published [51]. In order to predict dose-response in humans from animals for future vaccines, dose-response data from an existing vaccine in multiple species is required. The unavailability of these data may hinder attempts to develop an allometric scaling approach, therefore publishing of both clinical and pre-clinical dose-response studies is of great importance. Secondly, most of the doses were measured in viral particles, which may be a sub-optimal measure [52] as the infectious ratio, the number of viral particles per infectious unit, can vary between vaccines [15]. Therefore, the use of VP in measuring vaccine dose limits the comparisons in dose between different vaccines. Finally, when applying IS/ID modelling to define the dose-response curve, it is also possible that, whilst three dosing levels may be sufficient to theoretically define simple curves like a sigmoid function, this may not be a large enough number of doses to determine dose-response behaviour with an appropriate degree of certainty.

The strategies used to optimise vaccine dosing are likely to be suboptimal. There might exist mathematical descriptions of dose-response that are informative when choosing the various doses to use for a given construct in a given species which have not yet been identified. Indeed, both Darrah and Belovsky have shown that the highest dose was not the most effective dose for adenoviral vectored vaccination against *Leishmania* and non-adenovirus vectored vaccination against HIV [53,54], and yet the bias to choose the maximum safe dose remains among most vaccine developers.

New methods of vaccine dose optimisation need to be developed. Understanding adenoviral vaccine dose-response may be able to be achieved through reviewing and comparing historical dose-response data and combining these with mathematical modelling methods. This may aid in ensuring that the optimal dose for protection and safety is identified, while minimising the number of human and animal participants required to decide that dose.

## Figures and Tables

**Table 1:** The number of papers that included dose-response studies for each host species identified in the review.

Number of Papers (%)	Host	Paper References
21 (60%)	Mouse	[18,22,24,25,26,29,32,33,34,35,36,39,40,41,43,44,45,47,48,49,50]
9 (26%)	Human	[16,17,19,20,21,23,27,28,30]
2 (6%)	Monkey	[38,42]
2 (6%)	Rat	[37,45]
1 (3%)	Rabbit	[31]
1 (3%)	Cattle	[46]

**Table 2:** The number and percentage of papers that included dose-response data for each adenovirus serotype.

Human	Non-Human Primates
Ad5 (16, 46%) [22,24,25,29,31,33,34,38,39,42,43,44,45,46,49,50]	ChAd63 (3, 9%) [24,30,43]
Ad35 (9, 26%) [19,21,23,24,26,28,35,36,39]	ChAd3 (3, 9%) [16,17,24]
Ad26 (4, 11%) [26,27,32,37]	AdC6 (1, 3%) [41]
Ad6 (2, 6%) [38,47]	AdC7 (1, 3%) [40]
Ad28 (1, 3%) [24]	sAd11 (1, 3%) [24]
	sAd16 (1, 3%) [24]
	sAdv-36 (1, 3%) [48]
	ChAdOx1 (1, 3%) [20]

**Table 3:** The number and percentage of papers that included dose-response data for each immunological response type.

Number of Papers (%)	Response Type	Paper References
18 (51%)	Antibody	[16,17,20,21,22,23,25,26,27,28,31,33,36,39,40,42,45,48]
12 (34%)	T cell count	[16,20,21,26,27,28,30,32,36,38,42,47]
12 (34%)	CD8+ T cell count	[19,22,24,32,34,35,36,38,39,48,49,50]
11 (31%)	Virus Neutralisation Titre	[22,25,27,29,30,34,36,37,43,44,46]
4 (12%)	CD4+ T cell count	[19,32,35,38]
3 (9%)	CD8+ T Cell, IFN-y+ Percentage	[19,21,41]
3 (9%)	CD4+ T Cell, IFN-y+ Percentage	[19,21]
2 (6%)	CD4+ T Cell, TNF-a+ Percentage	[19,21]
2 (6%)	CD8+ T Cell, TNF-a+ Percentage	[21]
2 (6%)	CD4+ T Cell, IL-2+ Percentage	[19,21]
2 (6%)	CD8+ T Cell, Il-2+ Percentage	[21]
1 (3%)	CD4+ T Cell, Il-17+ Percentage	[19]

**Table 4:** The number and percentage of papers containing studies at each number of dosing levels.

Number of Papers (%)	Number of Dose Levels	Paper References
21 (60%)	3	[17,19,22,24,25,27,28,29,30,32,34,35,37,38,42,44,46,48,49,40]
8 (23%)	4	[16,20,21,23,26,36,40,43]
5 (14%)	5	[18,33,39,41,45]
1 (3%)	6	[31]
1 (3%)	7	[47]

## Appendix A

### Criteria A1. Search Terms, PubMed

**Strategy**	**PUBMED Search**	**#**
Concept 1 Adenovirus	adenovirus OR adenoviral OR adenovector OR adenovectors OR adenoviridae	1
Concept 2 Dose	dose OR doses OR dosage OR dosages OR dosing OR dosed OR dose response OR dose-response OR dose responses OR dose-responses OR dose response relationship OR dose-response relationship	2
Concept 3 Immune response	immunity OR immune OR immune-response OR immune response OR immune responses OR immune-responses OR immunostimulation OR immunodynamic OR immunodynamics OR immunisation OR immunisations OR immunization OR immunizations OR immunise OR immunises OR immunize OR immunizes OR immunised OR immunized OR immunising OR immunizing OR immunogenecity OR immunogenic OR immunology	3
Combine with AND	#1 AND #2 AND #3	
Add filter:	Humans, Other Animals, English	

### Criteria A2. Exclusion Criteria

A study was excluded if it; (a) was a gene transfer study, (b) was conducted in cancer models, (c) presented no immunological readouts, (d) used replication-competent adenovirus, (e) did not administer adenovirus via an intramuscular or subcutaneous route, (f) used adenovirus as a boost in a heterologous prime-boost vaccination regimen, (g) used adenovirus as a prime, in a heterologous prime-boost regimen, and did not report on immunological parameters post-prime and pre-boost, (h) presented a homologous dosing regimen with no reported immunological parameters after the initial dose, (i) only reported on immune parameters following a disease challenge, (j) co-administered an adjuvant, administered an adjuvant prior to adenovirus delivery or used an ad-juvant-encoded adenovirus vector, (k) only reported on pulmonary immunity to the adenovirus (l) presented only data on gene expression, (m) used a sample size of less than five mice per group, or less than three for non-human primates, (n) presented in-vitro derived data, or (o) was a systematic review or meta-analysis.
